# P-dipping of rice seedlings increases applied P use efficiency in high P-fixing soils

**DOI:** 10.1038/s41598-020-68977-1

**Published:** 2020-07-17

**Authors:** Aung Zaw Oo, Yasuhiro Tsujimoto, Njato Mickaël Rakotoarisoa, Kensuke Kawamura, Tomohiro Nishigaki

**Affiliations:** 1 Japan International Research Center for Agricultural Sciences, 1-1 Ohwashi, Tsukuba, Ibaraki 3058686 Japan; 2 Département de Recherche Rizicoles (DRR), Centre National de Recherche Appliquée au Développement Rural (FOFIFA), BP 1690, Tsimbazaza, Antananarivo, Madagascar

**Keywords:** Abiotic, Plant breeding

## Abstract

Applied phosphorus (P) use efficiency is generally low due to the low mobility of P in soil and its affinity to form insoluble complexes. Localized P application nearby the root zone is a potential approach to overcome this issue in crop production. However, the interaction with soil conditions is little understood, which results in less effective application of this approach. Using root-box experiments and changing P-retention capacity of soils, we revealed that applied P use efficiency of rice can be substantially improved by dipping seedlings in P-enriched slurry at transplanting (P-dipping) even in highly P-fixing soils. Spatial analysis of soluble P in soils indicated that P-dipping creates a P hotspot because the P-enriched slurry is transferred with seedling roots. The P hotspot could have induced vigorous surface root and facilitated further P uptake from the spot. In contrast, the effect of conventional P incorporation depended on P-retention capacity of soils; no increases in soluble P content in soils or plant P uptakes were observed when P-retention capacity was high. Our finding of significant interaction between localized P application and a specific soil property should help improving applied P use efficiency and achieving sustainable rice production against depleting P fertilizer resources.

## Introduction

Availability of phosphorus (P) is generally low in crop production systems relative to other nutrients due to its immobile nature and affinity to form insoluble complexes with other minerals in soil. Fertilizer P added to soils is promptly fixed by active Al and Fe or poor crystalline aluminosilicates, ferrihydrite, and Al- and Fe-humus complexes^1^. Moreover, soil P retention capacity is particularly high in volcanic soils or Andosols with large amounts of active Al and Fe oxides and in highly weathered soils in the tropics, e.g., Oxisols, Alfisols, Ultisols^2^. Such soils with high P retention capacity have low applied P use efficiency, that is, low crop P uptake relative to the amount of P applied. Fixen et al. summarized that the typical range of apparent fertilizer recovery efficiency for cereal crop production (maize, rice, and wheat) is merely 15 to 25% for P, much lower than that for nitrogen (N) at 40 to 65%^3^.

To overcome the low applied P use efficiency, large amounts of P have been continuously applied to obtain high grain yields in developed countries where crop and fiber productions largely rely on external mineral fertilizer inputs. For instance, the P application rate for lowland rice production in Japan averages from 52 to 105 kg P ha^−1^ depending on the soil type^4,5^, while the P uptake by grain and straw were estimated at merely 18 kg P ha^−1^ (using the average grain yield of 6 t ha^−1^ and a harvest index of 0.5). However, continuous application of excess amounts of P fertilizer in agricultural systems causes environmental problems such as the eutrophication of lakes and marine estuaries^6^. Moreover, given the finite nature of rock phosphate, from which P fertilizers are produced, and its increasing cost, it is vital to investigate availability of P in soil and strategies to improve applied P use efficiency. Such strategies are also critical in low-input systems, such as in rice production on smallholder farms in Sub-Saharan Africa (SSA), where rice yield is primarily restricted by P-deficient soils and inadequate P fertilizer inputs^7–9^.

Localized P application—the application of P fertilizer in the root zone, where P is readily accessible to the plants, instead of conventional broadcasting or incorporation—is one of the most studied options for improving applied P use efficiency^10,11^. A number of studies have demonstrated significant increases in grain yield and applied P use efficiency with localized P application for upland crops such as maize, pearl millet, sorghum, cowpea, and groundnut^12–16^. Positive effects of localized P application have been observed in direct-seeded upland and lowland rice production systems where small amounts of P are placed along with planting^17–19^. Root architectural responses of rice may also facilitate access to available P hot spots created by localized P application, and thus improve applied P use efficiency. For example, He et al. observed root architectural changes in upland rice, such as the formation of cluster-root-like fine roots and high root allocation to areas with high P concentrations, suggesting preferential root proliferation in P hot spots^20^.

In transplanted rice production systems, small amounts of P application to the nursery bed is one conventional approach to increase P use efficiency. A key function of this technique is to develop vigorous seedlings and accelerate initial growth after transplanting which in turn improve grain yields or alleviate post-transplanting stresses such as flooding and salt stresses^21–23^. However, this technique has a risk of P mining from soils even when large yield gains are achieved because additional nutrients transferred to the main fields with “vigorous seedlings” are negligible while the large proportion of applied P is retained in the nursery bed. Alternatively, dipping rice seedlings into P-enriched slurry (P-dipping) is more direct and localized P application method by placing a certain amount of P with slurry attached to seedling roots at transplanting and has little risk of P-mining^24^. While there have been few studies on this P-dipping technique, institutional reports and newsletters have noted that P-dipping results in yield increases of 10–50% or equivalent yields with a 40–60% reduction in P application rates relative to P broadcasting or incorporation^25–27^. Our recent study on P-dipping found that it is an effective technique to improve both applied P use efficiency and rice yield in smallholder farms in Madagascar^24^.

However, to date, little is known about how localized P application interacts with field conditions. This information is critical for farmer decision-making when it comes to this laborious technique. In particular, interactions with soil properties should be considered, such as soil available P concentration and P retention capacity.

Results from our recent field study imply that P-dipping might be more effective in soils with high P retention capacity^24^. Balasubramanian et al. also reported consistently higher yields with P-dipping relative to P-broadcasting in high P-fixing soils in the central highlands of Madagascar; however, no soil data were presented^28^. De Bauw et al. observed that P placement in the planting hole is more important when mobility of P is low, for example, in moist field conditions relative to irrigated fields^19^. Based on these preliminary results, we hypothesized that localized P application via P-dipping is more effective in high P-fixing soils or when soil P mobility is limited. This is because most of the broadcasted or incorporated P may become immobile and unavailable to plants in the high P-fixing soils, while a greater fraction of applied P may remain available in the root zone when P-dipping is used.

In the present study, a root-box experiment was conducted to compare biomass production and plant P uptake in rice as a response to three different P application methods—P-dipping (P_dip_), P incorporation (P_inco_), and no P (Ct)—with soils ranging in P retention capacity. Volcanic soil (VS) and red-yellow soil (YS) were used as typical high P-fixing soils, and the P retention capacities of both soils were artificially reduced by mixing them with granite sand (VS + Sand and YS + Sand). Then, additional experiments were conducted to identify the mechanisms contributing to the interaction between P application method and soil P retention capacity, that is, root architectural changes and spatial distributions of soluble P in soils.

## Results

### Interaction between P application method and soil P retention capacity on biomass production and plant P uptake (Experiment 1)

The physical and chemical properties of the experimental soils are summarized in Table 1. Briefly, the volcanic soil (VS) was a sandy loam with a pH of 5.7 and high concentrations of oxalate-extractable Al and Fe. The red-yellow soil (YS) was a clay loam with a pH of 7.8 and low concentrations of oxalate-extractable P (available P). The P retention capacities of VS and YS were 99 and 23%, respectively. By mixing with sand, their P retention capacities were reduced from 99 to 91% for VS and from 23 to 7% for YS.

**Table 1:** Physicochemical properties of experimental soils.

Parameters	Volcanic soil (VS)	Volcanic + sand (VS + Sand)	Red-yellow soil (YS)	Red-yellow soil + sand (YS + Sand)
pH (H_2_O)	5.7	7.0	7.8	8.1
EC (mS m^−1^)	4.4	8.6	14.2	20.4
Total N (g kg^−1^)^a^	1.8	0.9	0.7	0.4
Total C (g kg^−1^)^a^	45.5	23.8	12.8	8.3
C:N ratio	26.0	25.5	19.4	21.6
P retention (%)^b^	99.0	91.0	23.0	7.0
P_oxalate_ (mg kg^−1^)^c^	400.0	242.0	267.0	154.0
Al_oxalate_ (g kg^−1^)^c^	46.1	22.6	1.5	0.9
Fe_oxalate_ (g kg^−1^)^c^	25.7	13.4	3.6	2.2
Sand (%)^d^	83.4	–	41.2	–
Silt (%)^d^	10.5	–	23.7	–
Clay (%)^d^	6.1	–	35.1	–
Texture	Loamy sand	–	Clay loam	–

^a^NC analyzer, Sumigraph NC-220F (SCAS, Japan).

^b^The proportion of absorbed P after shaking 5 g of soil with 25 ml of 1,000 ppm P solution for 24 h.

^c^Inductively coupled plasma mass spectrometer (ICPE-9000, Shimazu, Japan) after oxalate extraction.

^d^Sieving and pipetting method.

**Figure 1:**
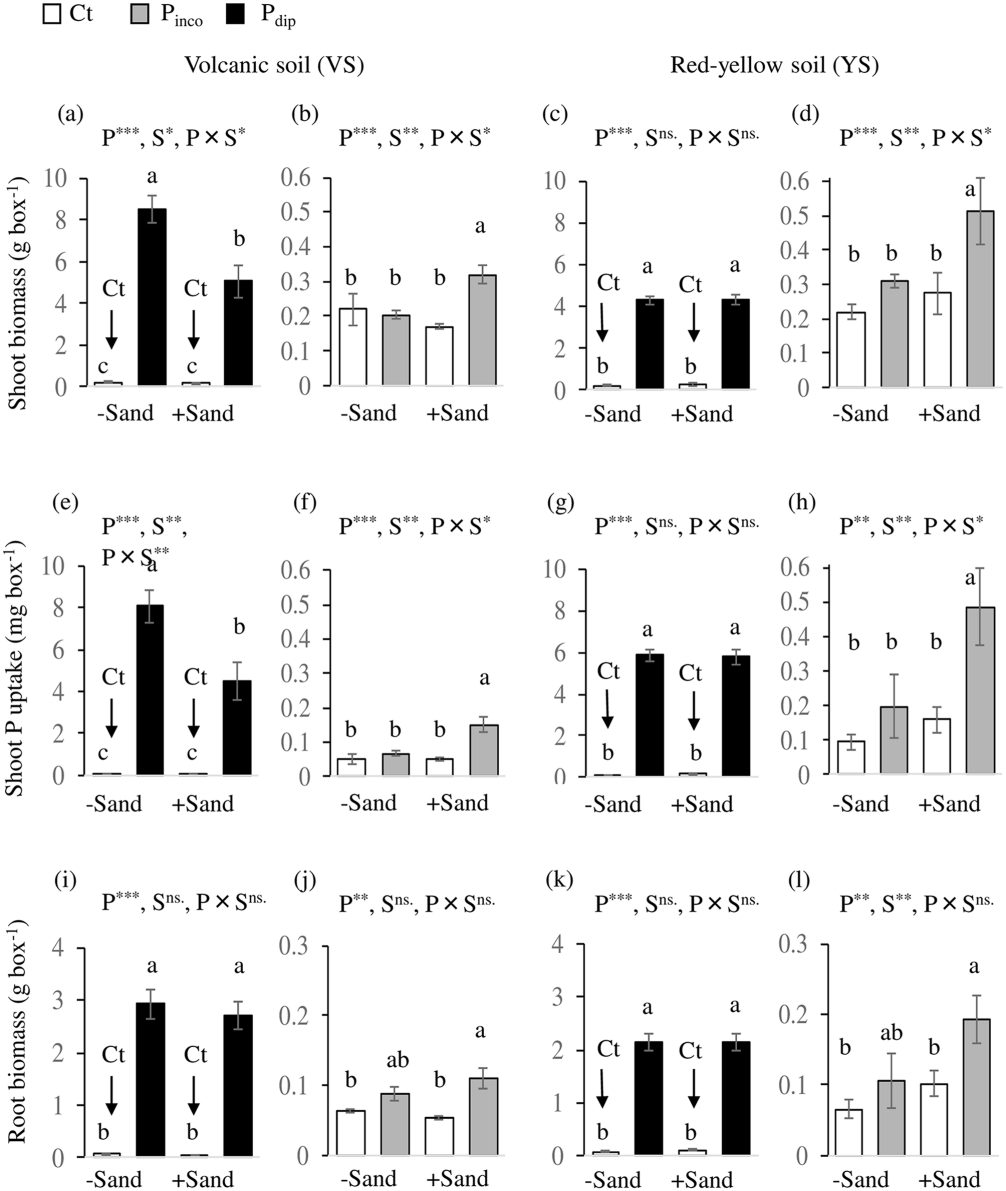
The effect of dipping seedlings in P slurry on shoot mass and shoot P content at 42 days after transplanting under different soil conditions (Experiment 1). Error bars indicate standard deviation. P_dip_: P-dipping, P_inco_: P incorporation, Ct: control. VS: Volcanic soil, YS: Red-yellow soil. (**a**), (**e**), (**i**): Ct vs. P_dip_ in VS. (**b**), (**f**), (**j**): Ct vs. P_inco_ in VS. (**c**), (**g**), (**k**): Ct vs. P_dip_ in YS. (**d**), (**h**), (**l**): Ct vs. P_inco_ in RS. *p < 0.05, **p < 0.01, ns: not significant at 5% level. P, S, and P × S indicate the effect of P treatment, sand incorporation, and their interaction, respectively.

The P_dip_ treatment substantially increased shoot biomass production relative to Ct from 0.22 to 8.52 g box^−1^ for VS, 0.17 to 5.07 g box^−1^ for VS + Sand, 0.22 to 4.30 g box^−1^ for YS, and 0.27 to 4.34 g box^−1^ for YS + Sand (Fig. 1a,c; Fig. S1). The effect of P_dip_ on shoot biomass was greater without sand incorporation for VS and equivalent between ‘ + Sand’ and ‘− Sand’ treatments for YS. Likewise, the shoot P uptake in P_dip_ was substantially increased by 8.09 mg box^−1^ for VS, 4.46 mg box^−1^ for VS + Sand, 5.87 mg box^−1^ for YS, and 5.78 mg box^−1^ for YS + Sand (Fig. 1e,g). The P_dip_ treatment achieved equivalent or greater increases in P uptakes even in the high P-fixing original soils without sand incorporation. Similar to shoot biomass, P_dip_ increased root biomass relative to Ct (Fig. 1i,k; Fig. S1).

On the other hand, shoot biomass and P uptake were not different between P_inco_ and Ct in the original soils, VS and YS, which is to say, P_inco_ had no effect on biomass production or shoot P uptakes under high P-fixing conditions (Fig. 1b,d,f,h; Fig. S1). However, P_inco_ significantly increased biomass production relative to Ct from 0.17 to 0.32 g box^−1^ for VS + Sand (Fig. 1b) and from 0.27 to 0.51 g box^−1^ for YS + Sand (Fig. 1d). Likewise, P_inco_ significantly increased shoot P uptake in soils with sand incorporation. These increases in shoot biomass and P uptakes in the ‘ + Sand’ treatment was more pronounced for YS than VS, whose P-fixing capacity was less affected by sand incorporation. No significant interaction between P (Ct vs. P_inco_) and sand treatments were observed for root biomass (Fig. 1j,l). The P_inco_ treatment slightly increased root biomass, even for the original soils of VS and YS; however, the effect was larger for soils mixed with sand.

### Effect of P application on the spatial distribution of soluble P (Experiment 2)

**Figure 2:**
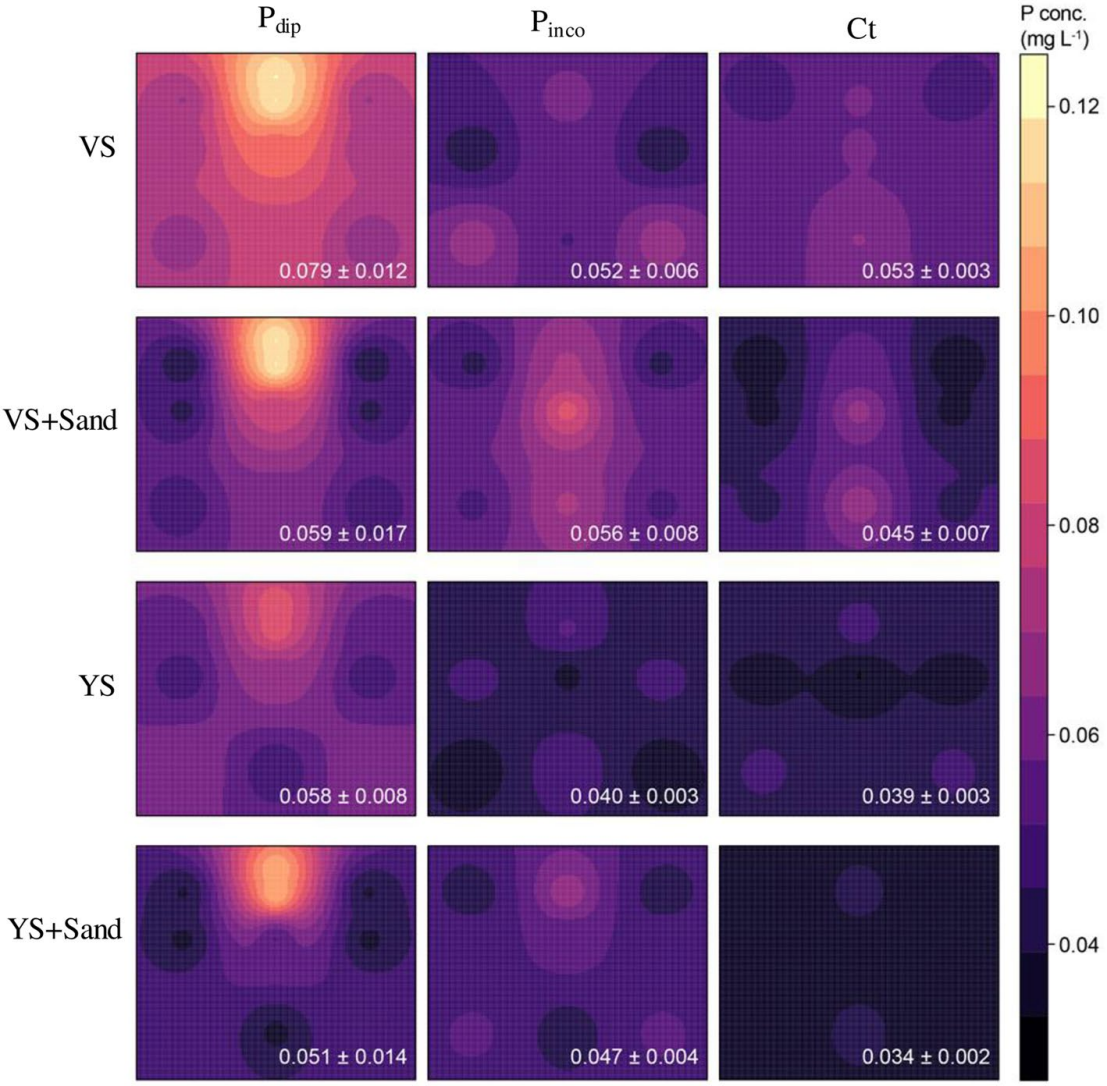
Spatial distribution of soluble P concentration in the soils of root boxes (Experiment 2). P_dip_: P-dipping, P_inco_: P incorporation, Ct: control. VS: Volcanic soil, YS: Red-yellow soil. The average (± standard deviation) soluble P concentration across the entire root box is given in white text.

Spatial analysis of soluble P in soils clearly showed hot spots of soluble P where P was locally applied to simulate P-dipping (Fig. 2). This spatial pattern was consistently observed for both VS and YS with and without sand incorporation. Conversely, no hot spots of soluble P were observed in either P_inco_ or Ct.

The average soluble P concentration across the entire root box was slightly higher in the original soils than those with sand incorporation. However, for all soils, the average soluble P concentration was higher in the P_dip_ treatment compared to P_inco_ and Ct. On the other hand, the average soluble P concentration did not differ significantly between the P_inco_ and Ct treatments, that is, P_inco_ did not increase soluble P in either VS or YS. The P_inco_ treatment increased average soluble P concentrations compared with Ct, when soil P-retention capacity was lowered by sand incorporation. As a result, the differences in average soluble P concentration between P_dip_ and P_inco._ were smaller with sand incorporation than in original soils.

### Root architectural changes in responses to different P application method (Experiment 3)

Experiment 3 produced equivalent shoot biomass and shoot P uptakes between P_dip_ and P_inco_ by amplifying the P application rates of P_inco_ from 90 mg P_2_O_5_ box^−1^ to 300 mg P_2_O_5_ box^−1^ (Table 2). For this experiment, only the VS and VS + Sand soils were used. Because there was no significant interaction between P application treatment and sand incorporation for any measured variables, the “ + Sand” and “− Sand” treatments were combined and means are shown in Table 2. While shoot biomass was the same, P_dip_ had significantly greater root biomass than P_inco_, and therefore had a higher root to shoot ratio (Table 2, Fig. S2). P_dip_ developed greater and wider nodal root systems than P_inco_, with nodal root number increasing by 23%, nodal root length by 16%, and root cone angle by 7%. Likewise, secondary branching degree, basal lateral root density, and relative root allocation to the topsoil layer (0–3 cm) were significantly greater in P_dip_ than P_inco_, by 26%, 37%, and 24%, respectively, indicating vigorous surface root development. The total root length in P_dip_ was greater than P_inco_ by 20%.

**Table 2:** Shoot and root architectural responses of rice to localized and conventional P application (Experiment 3).

	Shoot biomass (g box^−1^)	Shoot P uptake (mg P box^−1^)	Root biomass (g box^−1^)	Total root length (cm)	Nodal root length (cm)	Nodal root number	Basal lateral root density score	Secondary branching degree score	Root cone angle (°)	Relative root allocation to the topsoil layer
P_dip_	1.8a	2.69a	0.64a	7208a	2796a	95.9a	7.1a	3.9a	160.8a	0.21a
P_inco_	1.6a	2.73a	0.43b	6027b	2405b	77.8b	5.2b	3.1b	150.3b	0.17b
P	ns	ns	*	**	**	**	**	**	**	**
Sand (S)	ns	ns	*	**	**	**	**	**	**	ns
P × S	ns	ns	ns	ns	ns	ns	ns	ns	ns	ns

P_dip_: P-dipping (90 mg P_2_O_5_ box^−1^), P_inco_: P incorporation (300 mg P_2_O_5_ box^−1^).

*p < 0.05, **p < 0.01, ns = not significant at 5% level. Different letters within a column indicate significant differences between treatments at *p* < 0.05 using Tukey’s HSD test.

## Discussion

The present study clearly demonstrated that P-dipping can achieve high applied P use efficiency in transplanted rice, even in high P-fixing soils, compared to conventional incorporation of P. In order to achieve similar plant biomass and P uptake with incorporation, 300 mg P_2_O_5_ box^−1^ was needed compared with only 90 mg P_2_O_5_ box^−1^ for P-dipping. These results demonstrate that P-dipping is a potential strategy to overcome low applied P use efficiency in high P-fixing soils, and hence reduce the need for excess P application.

The effect of P incorporation was improved by lowering P retention capacity of soils by sand incorporation. This result also supports there exists significant interaction between P application method and P retention capacity of soils. Previous studies have shown that localized P application is more effective in P-deficient soils and less P-mobile conditions in millet^14^, lucerne^29^ and rice^18,19,24,28^. However, the present study is the first to show a significant interaction between P application method and soil P retention on plant biomass and P uptake. This finding will help inform farmers about where they should apply this slightly laborious localized P application technique effectively. Nishigaki et al. identified large variations in the P retention capacity and the amounts of oxalate-extractable Al and Fe (active Al and Fe) in soils in the central highlands of Madagascar, even among nearby lowland fields^30^. Their results and our finding imply that returns from P fertilizer inputs can be further improved by selectively using the P-dipping technique in fields with high P retention capacities.

The interaction between P application method and soil P retention capacity on aboveground biomass and P uptake corresponded to the spatial distribution of soluble P in soils (Fig. 2). The hotspot of soluble P as a result of P-dipping is in accordance with the P diffusion model of De Bauw et al. in which they assumed that larger proportions of applied P remain soluble with localized P application because the binding sites around the application points get saturated^19^. In the same mechanism, the P-dipping could have developed the P hotspot near the root zone even under highly P-fixing soils. Previous field experiments also detected soluble P hotspots as a result of localized P fertilizer application compared to uniform P incorporation^31,32^. Akahane et al. reported that high amount of P was remained available up to harvest within a relatively small area of 2 cm (vertical) by 4 cm (horizontal) from the application spot as a result of localized P application in continuously flooded lowlands^32^. Therefore, with the P-dipping technique, the P hotspot nearby the root zone consistently supplied P for rice growth, which in turn developed vigorous root systems. Then, the vigorous root systems facilitated further P uptakes from the soils. This positive feedback loop could have caused large differences in biomass and P uptakes between the P dipping and P incorporation treatments.

Contrarily, in the case of uniform P incorporation, all the applied P was apparently fixed in the original soils, which could have severely restricted both initial P uptakes and root development. Increased amounts of soluble P in reduced P-fixing conditions explain the responses of rice to P_inco_ with and without sand incorporation. This is in agreement with the general understanding of why applied P use efficiency is low in high P-fixing soils^33,34^.

The lower shoot P uptake and soil soluble P content with P-dipping in soil mixed with sand compared to original soil could be because (1) sand incorporation reduced soil fertility, not only of P but also of other minerals or (2) localized P was fixed of diffused away from the application point. According to De Bauw et al., changes in soil moisture from field capacity to saturated can enhance diffusion of locally applied P, resulting in lower soluble P within the root system^19^.

Changes in root architecture in response to P deficiency is well documented in rice and other plant species^6,20,35–37^. Our finding in Experiment 3 are in accordance with previous studies, showing preferential root allocation to soluble P hot spots. P-dipping accelerated root development and, in particular, development of surface root systems relative to P incorporation. P-dipping resulted in wider root cone angles and increased lateral root density and secondary branching degree. The same root architectural responses to P_dip_, i.e., wider in root cone angle and greater in lateral root density and secondary branching degree (root mass was substantially different between P_dip_ and P_inco_, though), were also observed for both soils in Experiment 1 (Table S1).

Wider root cone angles are, theoretically, a strategic response of rice when exposed to P deficiency. They result in greater root growth in the top layer of soil where P generally accumulates^38,39^. However, there was previously little empirical evidence for this in regard to localized P application or in submerged soil. The root architectural changes induced by P-dipping might have facilitated plant P uptake from the P hot spots in the soil surface. However, dense rooting “patches” or cluster-root-like fine roots, which have previously been observed in P hot spots^20,40^, were not present in our study. It should be noted that our experiment was conducted in small plastic boxes in which root development was spatially constrained. However, our observation of nonuniform distribution of soluble P in soils, preferential root architectural development, and aboveground plant responses are the first of its kind to explain the sequential mechanism of high P use efficiency of localized P application even in high P-fixing soils.

Further, the root morphological changes found in our study provide an opportunity to explore suitable varieties of rice that have ideal root systems for cultivation with localized P application. Specifically, applied P use efficiency may be further improved by combining P-dipping and shallow root varieties of rice. Recently, root architectural traits, such as steep root angle^41^, deep rooting^42^, and shallow rooting^43^, have been genetically identified in rice and the use of such quantitative trait loci in breeding activities has progressed. For instance, using a near isogenic line (DRO1-NIL) with a functional allele of *DRO1* that confers deep rooting, Uga et al. demonstrated that cadmium (Cd) accumulation in rice can be significantly reduced by altering root architecture as bioavailable Cd is present in upper or oxidized soils layers in paddy fields^38^. The potential interaction between genotypic variation in root architecture and localized P application need to be further explored.

## Conclusion

While localized P-application is a promising approach to overcome low applied P use efficiency in crop production, few studies have investigated the effect under different soil conditions. We demonstrated a significant interaction between P-dipping and soil P retention capacity, which was manipulated with sand incorporation, on the growth of rice. P-dipping substantially increased both plant biomass and P uptake irrespective of P retention capacity of soils, or even in the volcanic soil, which had very high P retention capacity. On the other hand, conventional incorporation of P into soil had little effect on soil soluble P, plant biomass, and P uptake of rice plants in high P-fixing soils. P incorporation had an effect on these factors only when the P retention capacity of the soil was reduced. Localized P placement indicated that P-dipping can create soluble P hot spots around the root system at transplanting, and plants with vigorous surface root systems, which might have facilitated P uptake from the P hot spots. These results not only provide evidence for a practical approach to overcome low P use efficiency in lowland rice production, but also provide information to help farmers select where to apply this slightly laborious technique depending on soil characteristics in order to maximize returns. Field experiments are required to confirm the interaction between P-dipping and P retention capacity of soils on rice yields.

## Materials and methods

### Soil and growing conditions

Experiments 1, 2, and 3 were conducted in a greenhouse with an automatic ventilation system at the Japan International Research Center for Agricultural Science (JIRCAS), Tsukuba, Japan. The daily mean temperature in the greenhouse throughout the experiments ranged from 25.0 to 34.2 °C (Thermo Recorder TR-50U2, T&D Corporation, Nagano, Japan).

Two different soils with contrasting P retention capacities were collected from the forest sub-soil layer (20–40 cm). The soils were a volcanic soil (VS) from a typical Andosol area in the Kanto district, Japan, and a typical red-yellow soil (YS) from Ishigaki Island, a subtropical island in Japan^44^. The soils were air-dried and passed through a 2.0 mm sieve. The soils were partly mixed with decomposed granite (+ Sand) at a ratio of 1:1 (w/w) to artificially reduce their P retention capacities. This resulted in four different soil condition: (1) VS, (2) YS, (3) VS + Sand, and (4) YS + Sand. The soils were thoroughly puddled in a bucket by adding NH_4_NO_3_ and K_2_SO_4_ and were filled into root boxes. The dimensions of the boxes were 30 cm height × 30 cm length × 3 cm width. They had a transparent acrylic sheet on one side, and soil was added to the boxes to a depth of 28 cm. The application rates of N and K_2_O were both adjusted to 220 mg box^−1^ by measuring the metric weight of each soil type in the boxes (2.52 L) after being puddled. The root boxes were put into 50-L containers with a height of 32 cm to maintain a consistent soil water level (4 cm water level) throughout the growing period and incubation for all the experiments.

### Experiment 1

Three P application methods—P-dipping (P_dip_), P incorporation (P_inco_), and no P application (Ct)—were factorially combined with the four soil conditions in a randomized complete block design with five replicates. For the P_dip_ treatment, a slurry with a P_2_O_5_ concentration of 4.3% (a mixture of 6.25 g triple super phosphate (TSP), 70 g air-dried soil, and 30 ml water) was prepared, in which seedling roots were dipped for 30 min before transplanting. The P concentration of the slurry and dipping duration were determined as optimal and most efficient based on our previous study^45^. The study identified that the P-dipping in the slurry with a range of P_2_O_5_ concentrations from 4.3 to 6.0% had an equivalent effect on initial biomass and P uptakes of rice plants while the higher concentration at 7.5% had a risk of seedling damage because of chemical injuries to rice seedlings. With the 4.3% P_2_O_5_ slurry, the amount of P transferred at transplanting (slurry attached to seedling roots) was estimated at approximately 90 mg P_2_O_5_ box^−1^. For the P_inco_ treatment, TSP was added with N and K at puddling at the same application rate of 90 mg P_2_O_5_ box^−1^.

Then, 21-day old seedlings of cv. IR 64 (*Oryza sativa* L.) were transplanted with one seedling per box on July 25, 2019 and grown for 42 days after transplanting (DAT). At 42 DAT, plant shoots were removed at ground level and oven-dried at 70 °C for > 48 h to determine shoot dry weight. Shoot P concentration was measured with the molybdate blue method^46^ after dry ashing at 550 °C for 2 h and digesting with 0.5 M HCl. Shoot P uptake (mg box^−1^) was calculated by multiplying the P concentration by the dry weight. Root architectural parameters and root mass were determined in the same manner as detailed in Experiment 3.

### Experiment 2

**Figure 3:**
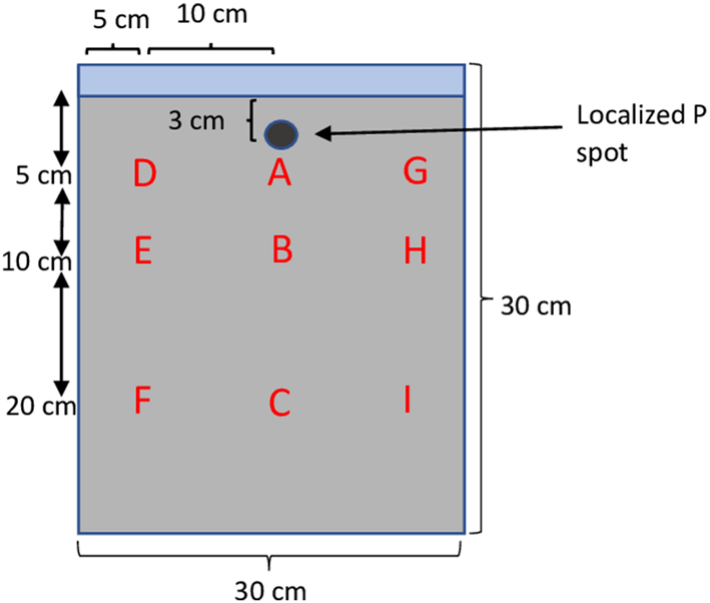
Schematic representation of a root box (30 cm × 30 cm × 3 cm) with soil water solution sampling points A to I. Soil solution samplers were installed in root boxes and soil water solutions were sampled at weekly intervals from the points A–F assuming that the horizontal distribution in the root box was symmetric.

Additional complete sets of root boxes—factorial combinations of the four soil types and three P application methods with four replicates—were established beside Experiment 1 to repeatedly sample and analyze the P content in soil solutions under flooded and uncultivated conditions. Soil solution samplers attached to ceramic tubes were installed in six different positions in each root box; at 5 cm, 10 cm, and 20 cm depths both in the center (Position A, B, and C in Fig. 3) and at 5 cm from one side (Position D, E, and F in Fig. 3). Nutrients were applied in the same way as Experiment 1, except for the P_dip_ treatment in which 90 mg P_2_O_5_ was dissolved in 20 ml water and injected into the soil at a depth of 3 cm in the center of the root box using a 50-ml syringe to simulate the localized P application in the P-dipping treatment. All boxes were put in a large container and kept under flooded conditions for 42 days as with Experiment 1. Soil water samples were collected at 7, 14, 21, 28, 35, and 42 days after flooding using a 50-ml syringe.

Collected soil water samples were analyzed for soluble P concentration using the Malachite green method^47^. The average of four replicates at 7 and 14 days after flooding were used for the spatial analysis because the data varied considerably at the other sampling dates. The vertical spatial distribution of soluble P concentration was estimated by interpolating on nine positions (A–I in Fig. 3) with the inverse distance weighting (IDW) method using R software (ver. 3.6.2)^48^. The soluble P values for positions D–F were used for the positions G–I, assuming that the horizontal distribution in the root box was symmetric.

### Experiment 3

Experiment 3 was conducted to assess the effect of P application method on root architecture under similar levels of shoot biomass and P content. The soil and nutrient preparations and rice cultivation were conducted in the same manner as in Experiment 1, with a few exceptions. The P_2_O_5_ application rate for P_inco_ was increased to 300 mg box^−1^. For this experiment, only the VS and VS + Sand soils were used due to the shortage in available amounts of YS soils. The 21-day old seedlings of cv. IR 64 (*Oryza sativa* L.) were transplanted with one seedling per box on Sep 11, 2019. Shoots were sampled at 28 DAT and analyzed in the same manner as Experiment 1.

Root development was photographed using the pin-board method of Kano-Nakata et al.^49^ with a slight modification. Briefly, roots were pinned with a 5 mm mesh net and pinboard after which soils were washed off and digital images were taken. Then, root samples were collected to determine various root architectural traits and dry matter. Relative root allocation to the topsoil layer (0 to 3 cm), and root cone angle—the angle between the two most external left and right nodal roots to the vertical axis^50^—were determined using the digital images of root development and ImageJ (Version 1.52a, NIH, USA). The relative root allocation to the topsoil layer was calculated in the following equation;

$$\text{Relative}\;\text{root}\;\text{allocation}\;\text{to}\;\text{the}\;\text{topsoil}\;\text{layer}=\frac{\text{Fractional}\;\text{root}\;\text{coverage}\;\text{in}\;\text{the}\;\text{topsoil}\;\text{layer} \;(\%)\times \text{Area}\;\text{of}\;\text{topsoil}\;\text{layer} \left(3\,\text{cm} \times 30\,\text{cm}\right)}{\text{Fractional}\;\text{root}\;\text{coverage}\;\text{of}\;\text{the}\;\text{whole}\;\text{box} \left(\%\right)\times \text{Area}\;\text{of}\;\text{whole}\;\text{box} \left(30\,\text{cm} \times 30\,\text{cm}\right)}$$

Here, the fractional root coverage in the topsoil layer was estimated by the following procedure using ImageJ software; (1) The topsoil layer (3 cm in depth by 30 cm in length) was selected from the digital image of root development; (2) The root pixel in the selected area was extracted by adjusting the brightness of the Color Threshold command; (3) The fractional root coverage was calculated as the number of root pixels divided by the total number of pixels in the selected area using the Analyze Particles command.

The number of nodal roots were manually counted. Nodal root thickness score, basal lateral root density, and secondary branching degree were determined following De Bauw et al.^51^. Basal lateral root density—the spacing of lateral S-type branches^52^ at the nodal root base (from stubble up to 15 cm depth)—was scored using the shovelomics scoreboard (scores from 1 to 9 represent very sparse to highly dense lateral roots). The secondary branching degree—the degree of higher order root branching on L-type roots^52^ evaluated over the whole root system—was scored visually (scores from 1 to 5 represent very low (almost no branching) to very high branching). Nodal root length (cm box^−1^) and lateral root length (cm box^−1^) were determined based on root diameters using WinRhizo Pro (Regent Instrument, Quebec, Canada). Root diameters > 2 mm were discarded, as this is larger than the nodal root size, roots with a diameter < 0.2 mm were considered lateral roots^53^, and roots with a diameter between 0.2 and 2 mm were considered nodal roots.

### Statistical analysis

The data from Experiment 1 were analyzed using two-way analyses of variance (ANOVAs) to assess the single and interaction effects of P treatment (P_dip_ vs. Ct and P_inco._ vs. Ct) and sand incorporation treatment (− Sand vs. + Sand) for each soil type on the measured variables using XLSTAT 2019 (Addinsoft SAS). The replicates were treated as a random factor. The treatment means were compared at 5% level of probability using Tukey’s HSD test. Likewise, the data from Experiment 3 were analyzed using two-way ANOVAs to assess the single and interaction effects of P treatment (P_dip_ vs. P_inco_) and sand incorporation treatment (− Sand vs. + Sand).

## Supplementary information

Supplementary Information 1.
