# Supplementary material for: P-dipping of rice seedlings increases applied P use efficiency in high P-fixing soils
Source: Sci Rep. 2020 Jul 17;10:11919. doi: 10.1038/s41598-020-68977-1 (PMC7368074; doi:10.1038/s41598-020-68977-1)
Supplement: Supplementary file 1 — Supplementary Information 1. [file 41598_2020_68977_MOESM1_ESM.pdf]

P-dipping of rice seedlings increases applied P use efficiency in high P-fixing soils

Aung Zaw Oo[1], Yasuhiro Tsujimoto[1, *], Njato Mickaël Rakotoarisoa[2], Kensuke Kawamura[1], Tomohiro Nishigaki[1]

[1]Japan International Research Center for Agricultural Sciences, 1-1 Ohwashi, Tsukuba, Ibaraki 3058686, Japan

[2]Centre National de Recherche Appliquée au Développement Rural (FOFIFA), Département de Recherche Rizicoles (DRR), BP 1690, Tsimbazaza, Antananarivo, Madagascar

*Corresponding author. Tel/Fax: +81 29 838 6367; E-mail address: tsjmt@affrc.go.jp (Yasuhiro Tsujimoto)

Supplementary information

Table S1 Effect of P application method on root development at 42 days after transplanting under different soil conditions (Experiment 1). VS: Volcanic soil, YS: Red-yellow soil, P_{dip}: P-dipping, P_{inco}: P incorporation, Ct: control (no P application).

Fig. S1 Effect of P application method on shoot growth and root development of rice under different soil conditions at 42 days after transplanting (Experiment 1). P_{dip}: P-dipping (90 mg P_2O_5 box^{-1}), P_{inco}: P incorporation (90 mg P_2O_5 box^{-1}), Ct – no P application.

Fig. S2 Effect of P application method on shoot growth and root development of rice at 28 days after transplanting (Experiment 3). P_{dip}: P-dipping (90 mg P_2O_5 box^{-1}), P_{inco}: P incorporation (300 mg P_2O_5 box^{-1}).

Table S1 Effect of P application method on root development at 42 days after transplanting under different soil conditions (Experiment 1). VS: Volcanic soil, YS: Red-yellow soil, P_{dip}: P-dipping, P_{inco}: P incorporation, Ct: control (no P application).

		Total root length (cm)	Nodal root length (cm)	Lateral root length (cm)	Nodal root number	Basal lateral root density score	Secondary branching degree score	Root cone angle (°)
VS	P_{dip}	21796 a	8046 a	13750 a	221.5 a	7.9 a	4.3 a	143.7 a
	P_{inco}	6391 b	1623 b	4767 b	27.3 b	2.1 b	2.0 b	122.3 b
	Ct	3071 b	512 b	2559 b	23.0 b	2.0 b	1.7 b	128.0 b
YS	P_{dip}	25474 a	8033 a	17441	186.0 a	7.1 a	4.1 a	143.5a
	P_{inco}	4999 b	1153 b	3846	38.9 b	4.3 b	2.6 b	131.4 b
	Ct	3514 b	727 b	2787	22.6 b	3.0 b	2.2 b	123.9 b

The average over +Sand and -Sand were shown because of no interaction effect between P application methods and sand incorporation. Within each soil type, different letters indicate significant differences between treatments at $p < 0.05$ using Tukey's HSD test.

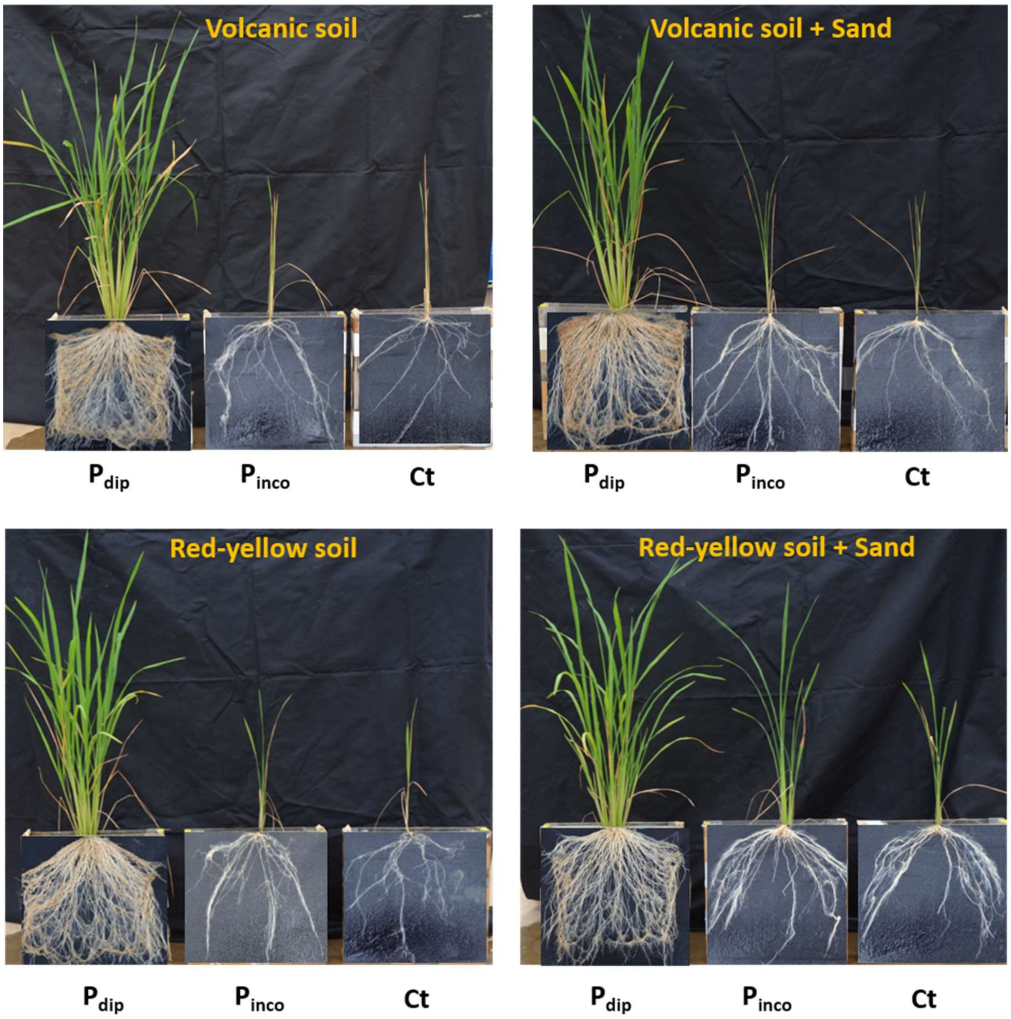

Fig. S1 Effect of P application method on shoot growth and root development of rice under different soil conditions at 42 days after transplanting (Experiment 1). P_{dip}: P-dipping (90 mg P_2O_5 box^{-1}), P_{inco}: P incorporation (90 mg P_2O_5 box^{-1}), Ct – no P application.

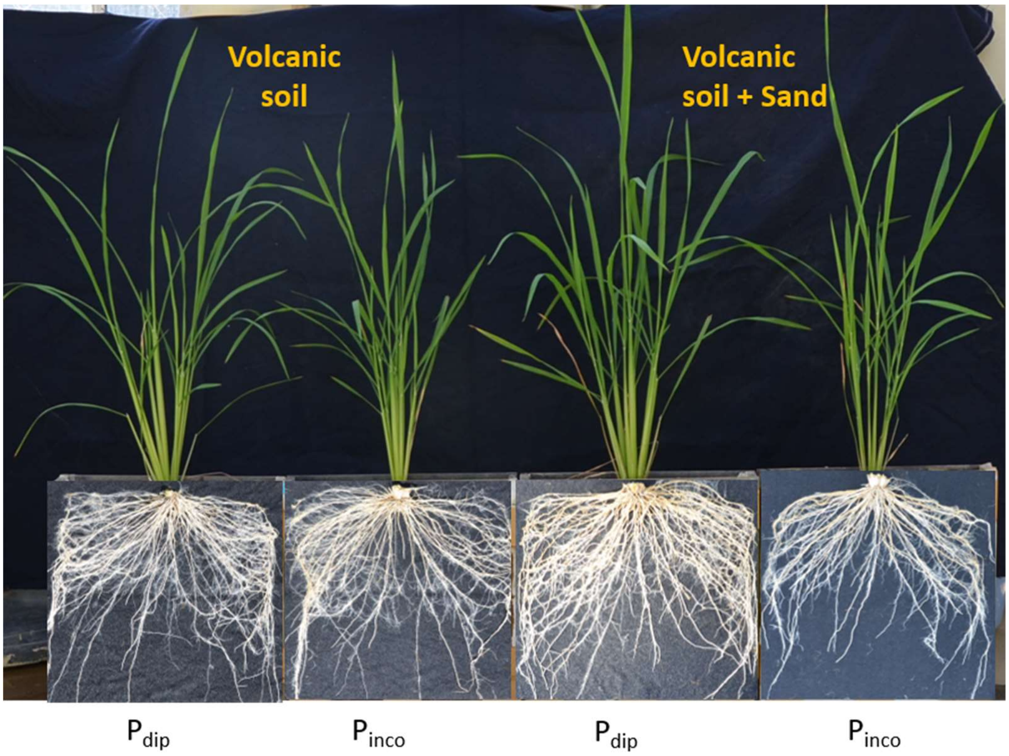

Fig. S2 Effect of P application method on shoot growth and root development of rice at 28 days after transplanting (Experiment 3). P_{dip}: P-dipping (90 mg P_2O_5 box^{-1}), P_{inco}: P incorporation (300 mg P_2O_5 box^{-1}).
